# Supplementary material for: Multimodal gene and targeted drug therapy for chronic myelogenous leukemia using BIM/MCL-1 chimeric nanoparticle: computational target analysis and therapeutic validation
Source: Nano Converg. 2026 Apr 13;13:13. doi: 10.1186/s40580-026-00543-3 (PMC13057112; doi:10.1186/s40580-026-00543-3)
Supplement: Supplementary file 1 — Supplementary Material 1 [file 40580_2026_543_MOESM1_ESM.pdf]

Multimodal gene and targeted drug therapy for chronic myelogenous leukemia using BIM/MCL-1 chimeric nanoparticle: Computational target analysis and therapeutic validation

Margaret L. Lugin[1†], Winnie Lei[2,3†‡], Rebecca T. Lee[4†], Jee Young Chung[5], Nicholas M. Katritsis[2,6], Woochang Hwang [2¶], Angela G. Fleischman[7*] Namshik Han[2,8,9,10,11,12*], Young Jik Kwon[1,4,5,13*]

[1]Department of Chemical and Biomolecular Engineering, University of California, Irvine, CA 92697, United States. [2]The Milner Therapeutics Institute, University of Cambridge, Cambridge, United Kingdom, CB2 0AW. [3]Department of Surgery, University of Cambridge, Cambridge, United Kingdom, CB2 0QQ. [4]Department of Biomedical Engineering, University of California, Irvine, CA 92697. [5]Department of Pharmaceutical Sciences, University of California, Irvine, CA 92697. [6]Department of Chemical Engineering and Biotechnology, University of Cambridge, Cambridge, United Kingdom, CB3 0AS. [7]Division of Hematology/Oncology, Department of Medicine, University of California, Irvine, CA 92697. [8]Cambridge Centre for AI in Medicine, Department of Applied Mathematics and Theoretical Physics, University of Cambridge, Cambridge, United Kingdom, CB3 0WA. [9]Cambridge Stem Cell Institute, University of Cambridge, Cambridge, United Kingdom, CB2 0AW. [10]Department of Quantum Information, Institute for Convergence Research and Education in Advanced Technology and Engineering, Yonsei University, Seoul, Republic of Korea, [11]Department of Nano Biomedical Engineering (NanoBME), Advanced Science Institute, Yonsei University, Seoul, Republic of Korea, [12]Center for Nanomedicine, Institute for Basic Science (IBS), Seoul, Republic of Korea,[13]Department of Molecular Biology and Biochemistry, University of California, Irvine, CA 92697.

[†]These authors equally contributed to the work.
[‡]Current address: Avatrial Ltd., Cambridge, United Kingdom, CB2 3QJ.
[¶]Current address: CardiaTec Bioscience Ltd., Cambridge, United Kingdom, CB2 1GE.
***Corresponding authors:** agf@uci.edu; nh417@cam.ac.uk; kwonyj@uci.edu

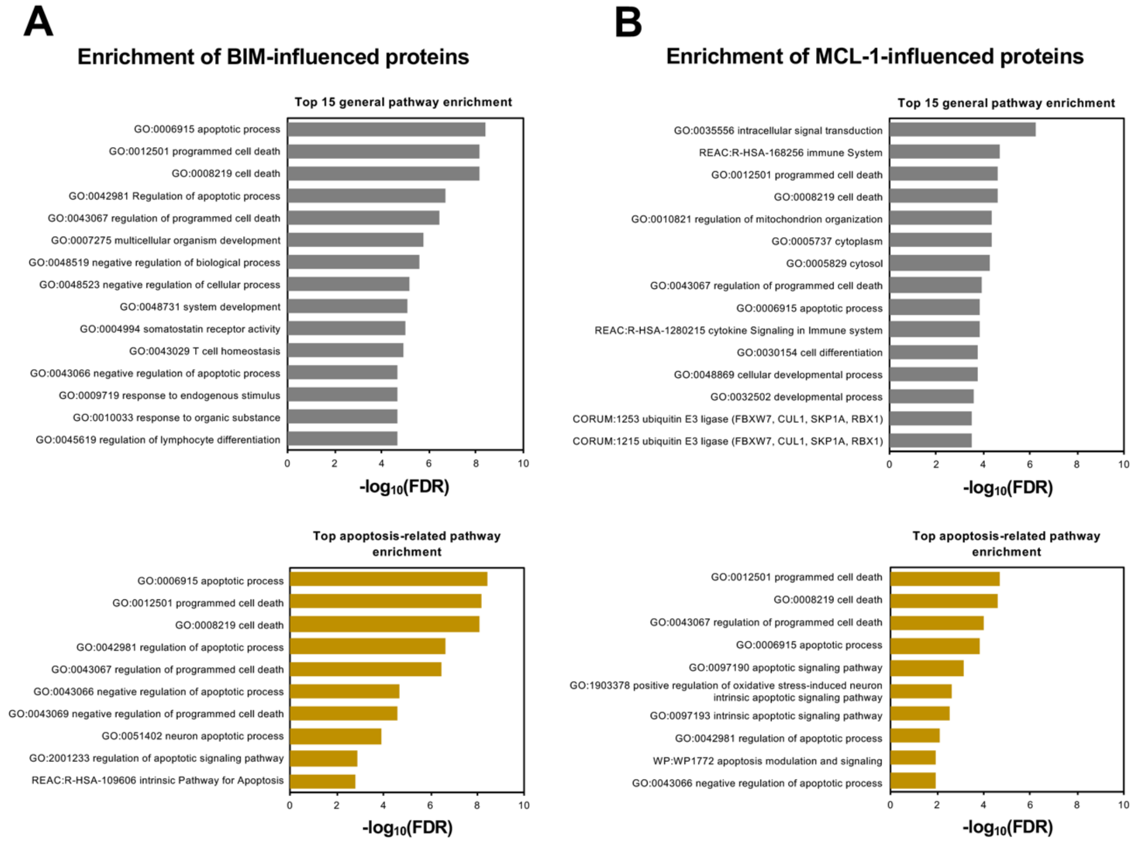

Figure S1. Enrichment of BIM-influenced and Mcl1-influenced proteins. Bar plots showing the -$\log_{10}$(False Discovery Rate) (FDR) of the top 15 pathways (top panel) and apoptosis-related pathways (bottom panel) enriched by the (A) BIM-influenced proteins identified from BIM Random Walk Restart (RWR) and (B) MCL-1-influened proteins from MCL-1 RWR in the CML PPI network. The high $\log_{10}$(FDR) of the apoptosis-related pathway suggests a large number of apoptotic-related proteins have been removed in CML BIM KI MCL-1 KO network, thus reducing the apoptosis pathway enrichment during BIM KI/MCL-1 KO ChNPs simulation.

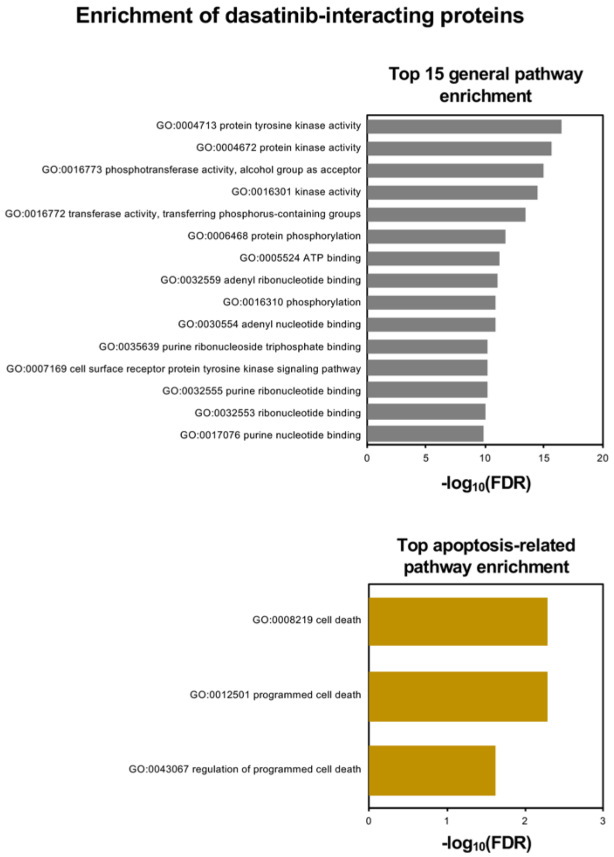

Figure S2. Enrichment of Dasatinib interacting proteins. Bar plots showing the -$\log_{10}$(False Discovery Rate) (FDR) of the top 15 general pathways (top panel) and apoptosis-related pathways (bottom panel) enriched by Dasatinib interacting protein. The high $\log_{10}$(FDR) of the apoptosis-related pathway suggests the removal of apoptotic-related proteins in CML BIM KI MCL-1 KO Dasatinib network, thus reducing the apoptosis pathway enrichment during BIM KI/MCL-1 KO ChNPs + Das simulation.

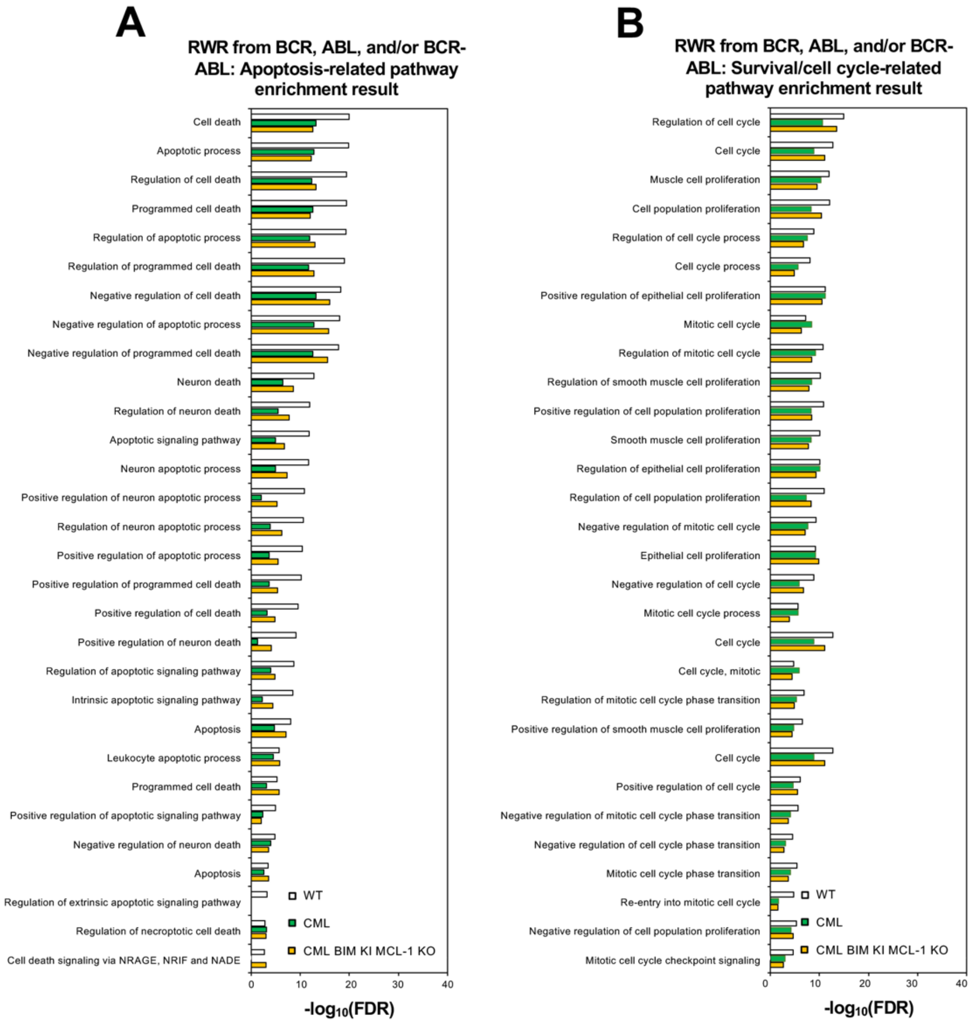

Figure S3. The effect of BIM KI and MCL-1 KO on BCR/ABL pathway activity in CML. Bar plots showing the enrichment of (A) apoptosis-related pathways and (B) survival or cell-cycle related pathways based on the RWR score seeded from BCR-ABL and/or BCR and ABL in wild-type (WT; white), CML (green), and CML treated with BIM KI and MCL-1 KO (yellow) networks. CML BIM KI MCL-1 KO demonstrates a mild restoration of apoptosis, survival, and cell cycle-related pathway activities that were lost from WT in the CML network.

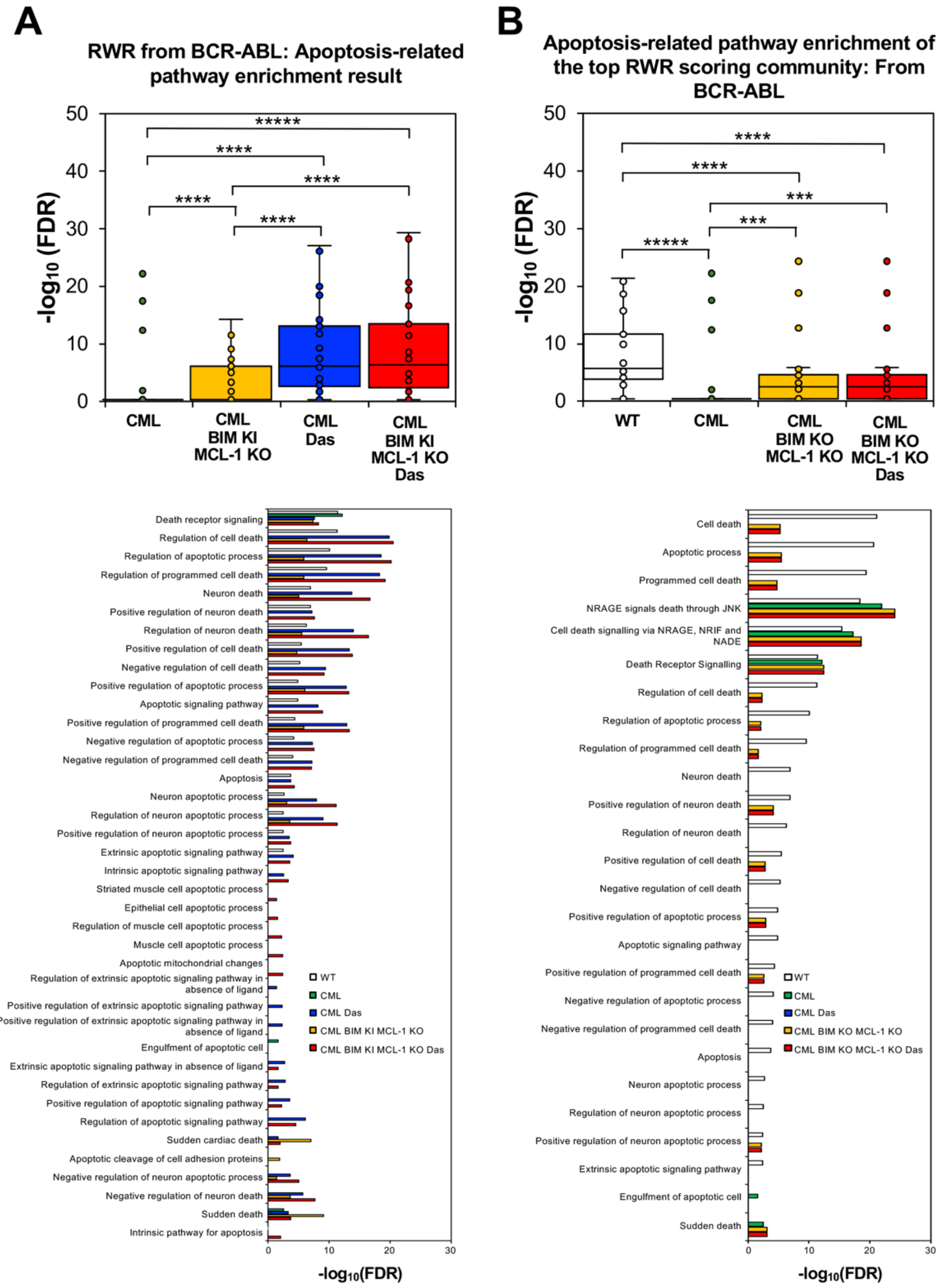

Figure S4. The effect of targeting BIM and MCL-1 on BCR-ABL signaling based on the community with highest RWR scores. Summary box plots (top panel) and bar plots (bottom panel) showing the enrichment of apoptosis-related pathways based on the highest RWR scoring community nodes seeded from BCR/ABL and/or BCR and ABL in (A) CML (green), CML treated with BIM KI and MCL-1 KO (yellow), CML treated with Dasatinib (blue), and CML treated with BIM KI and MCL-1 KO and Dasatinib (red) networks and (B) WT (white), CML (green), CML treated with BIM KO MCL-1 KO (yellow) and CML treated with BIM KO MCL-1 KO and Dasatinib (green). CML treated with BIM KI and MCL-1 KO with/without Dasatinib displayed increased apoptotic pathway enrichment compared to CML and other simulated treatments. (*: *p*-value ≤ 0.05; **: *p*-value ≤ 0.01; ***: *p*-value ≤ 0.001; *****: *p*-value ≤ 0.00001)

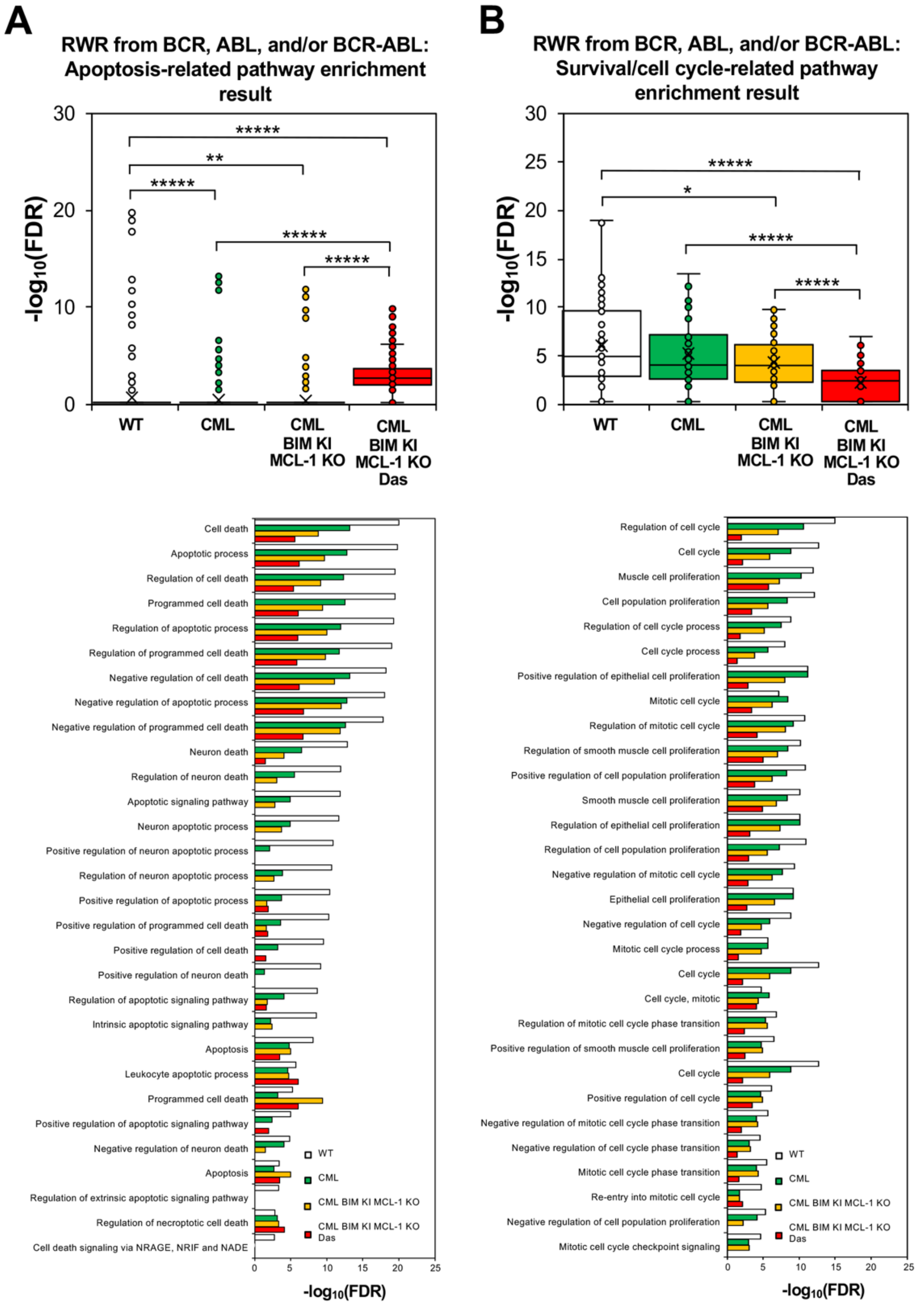

Figure S5. The effect of BIM KO and MCL-1 KO in CML. Summary box plots (top panel) and bar plots (bottom panel) showing the enrichment of (A) apoptosis-related pathways and (B) survival or cell-cycle related pathways based on the RWR score seeded from BCR-ABL and/or BCR and ABL in wild-type (WT; white), CML (green), CML treated with BIM KO and MCL-1 KO (yellow) and CML treated with BIM KO and MCL-1 KO with Dasatinib (red) networks. CML BIM KO MCL-1 KO with or without Dasatinib demonstrates a lower level of apoptosis, survival, and cell cycle-related pathway activities than CML or WT networks. (*: *p*-value ≤ 0.05; **: *p*-value ≤ 0.01; ***: *p*-value ≤ 0.001; *****: *p*-value ≤ 0.0001)

A The structure of the simulated heterogeneous multi-layer networks

Network	No. of nodes	No. of edges	No. of edges between KEGG and PPI layers
WT	189	141,502	14,834
CML	188	136,910	14,617
CML BIM KI MCL-1 KO	189	138,085	14,615
CML Das	188	135,329	14,493
CML BIM KI MCL-1 KO Das	189	136,504	14,491
CML BIM KO MCL-1 KO	188	134,354	14,438
CML BIM KO MCL-1 KO Das	187	131,777	14,256

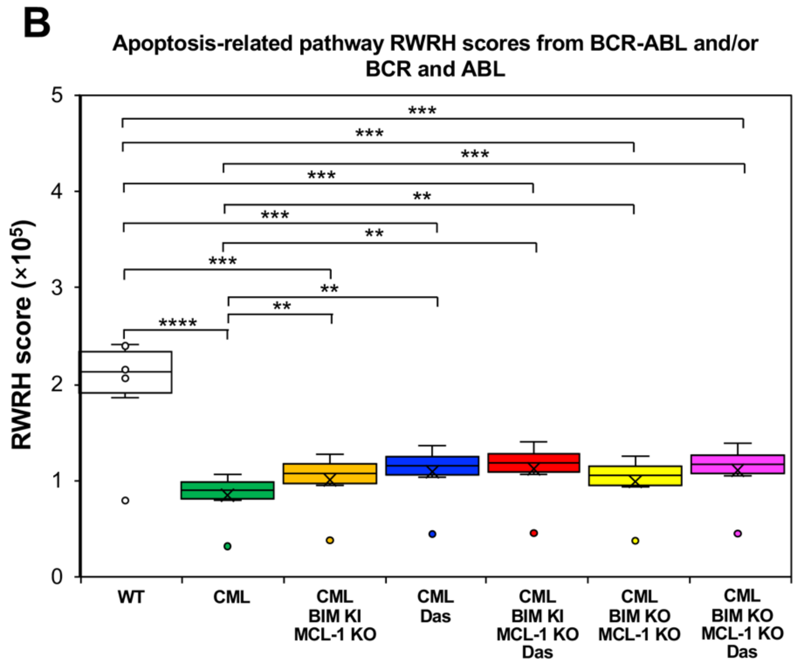

Figure S6. RWRH z-scores of multi-layer network with KEGG layer generated based on pathway enrichment results. (A) Table shows the total number of pathway terms in nodes and the number of edges connecting the KEGG pathway terms in the heterogeneous multi-layer network. The number of edges connecting the KEGG layer network to the PPI network is also summarized. (B) Box plot showing the Random-Walk-Restart-Heterogenous (RWRH) z-scores of all apoptosis-related pathways at the KEGG layer network, seeded from BCR-ABL and/or BCR and ABL at the PPI layer. Apoptosis-related pathways are shown to be significantly different between WT network and all CML networks included those under treatment. Apoptosis-related pathways also scored significantly lower to CML networks under any treatment. (**: p-value ≤ 0.01; ***: p-value ≤ 0.001; ****: p-value ≤ 0.0001)

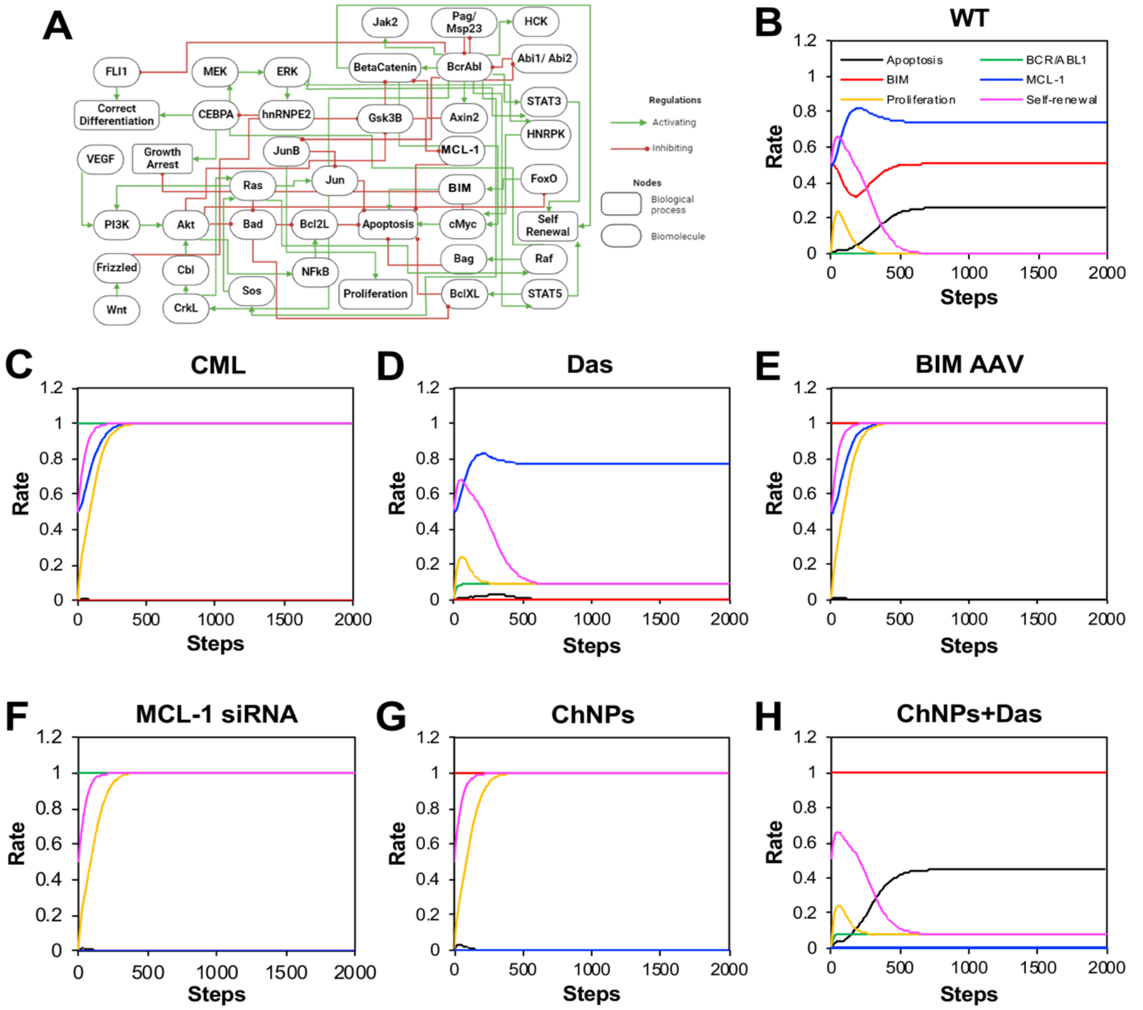

Condition	Average convergence steps	Fixed point (%)	Cycle (%)	No. of attractors	Dominant attractor(%)
WT	1013.60	1.60	98.40	113809	21.80
CML	1011.41	1.31	98.69	97730	22.00
Dasatinib	1012.99	1.52	98.48	108277	20.37
ChNPs	1011.08	1.28	98.72	93968	22.57
Das + ChNPs	1012.87	1.51	98.49	105248	20.00

Figure S7. CML Boolean network simulation. (A) The final reduced CML Boolean network topology. A green line with an arrowhead represents positive regulation, whereas a red line with a diamond indicates negative regulation. Activation frequencies for three pathway nodes, including Proliferation, Apoptosis, and Self-Renewal, together with three biomolecule nodes, including BCR-ABL, MCL-1, and BIM, were observed in simulated (B) WT, (C) CML, (D) CML treated with Dasatinib, (E) CML treated with BIM AAV, (F) CML treated with MCL-1 siRNA, (G) CML treated with BIM KI MCL-1 KO ChNPs, and (H) CML treated with BIM KI MCL-1 KO ChNPs and Dasatinib conditions. In Table, sensitivity analysis was performed on 2,000 steps and by sampling 5,000 random initial states for each condition. The model showed strong robustness to update-step variation: Convergence times remained consistent (average ≈1012 steps) and the overall attractor structure was stable across step limits. The Boolean model was sensitive to initial-state variation with each condition producing a large number of diffing attractors with a dominant attractor capturing ~21% of trajectories.

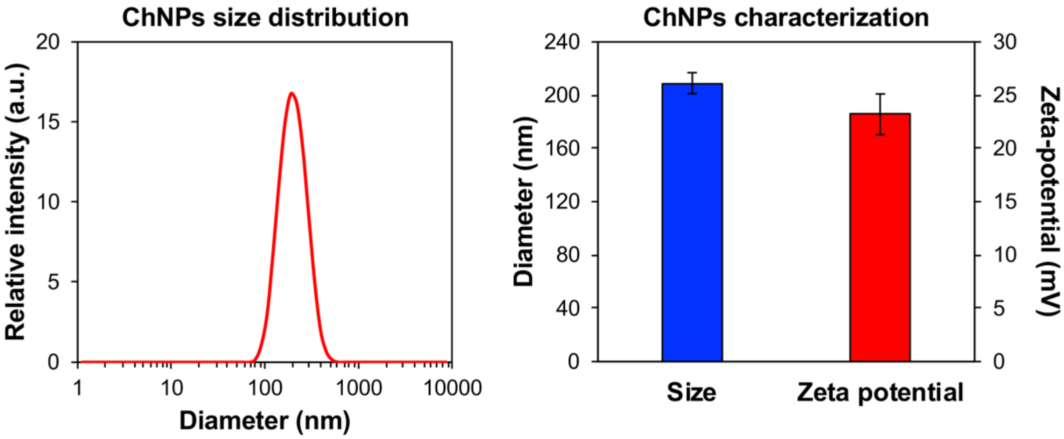

Figure S8. ChNP size and zeta potential characterization. ChNPs had a uniform size around 200 nm in diameter (PDI: 0.299 ± 0.042) as observed by the normal curve. Their zeta-potential was positive, as expected due to positive nature of the polymer shell being created. The encapsulation of AAV and siRNA was approximately ~100% as previously reported (Cho & Kwon, Biomaterials 33, 3316-3323, 2012).

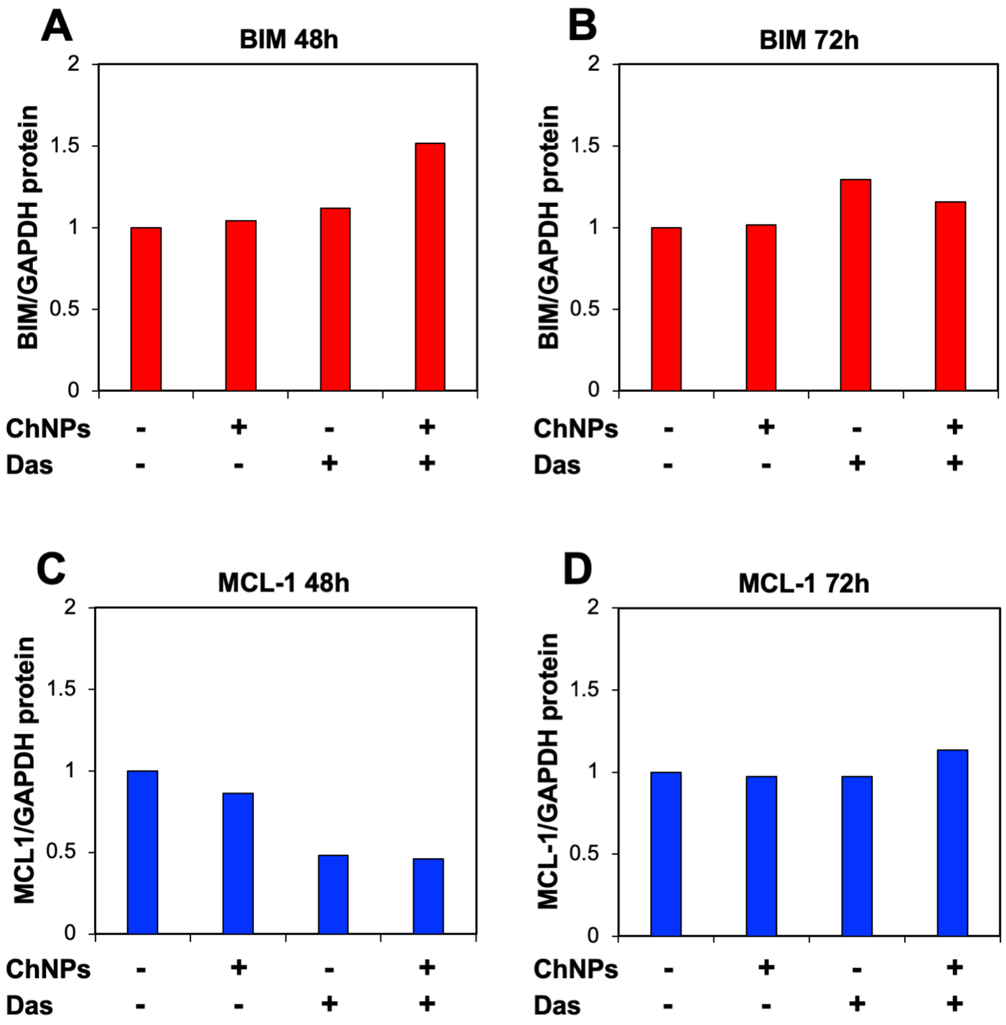

Fig S9. Western blot densitometric quantification normalized to GAPDH. Densitometric quantification of BIM and MCL-1 expression levels at 24 and 48 h after incubation with BIM/MCL-1 ChNPs and dasatinib. All bands were normalized to GAPDH as a loading control and Data are expressed as fold change relative to the untreated control group**.**

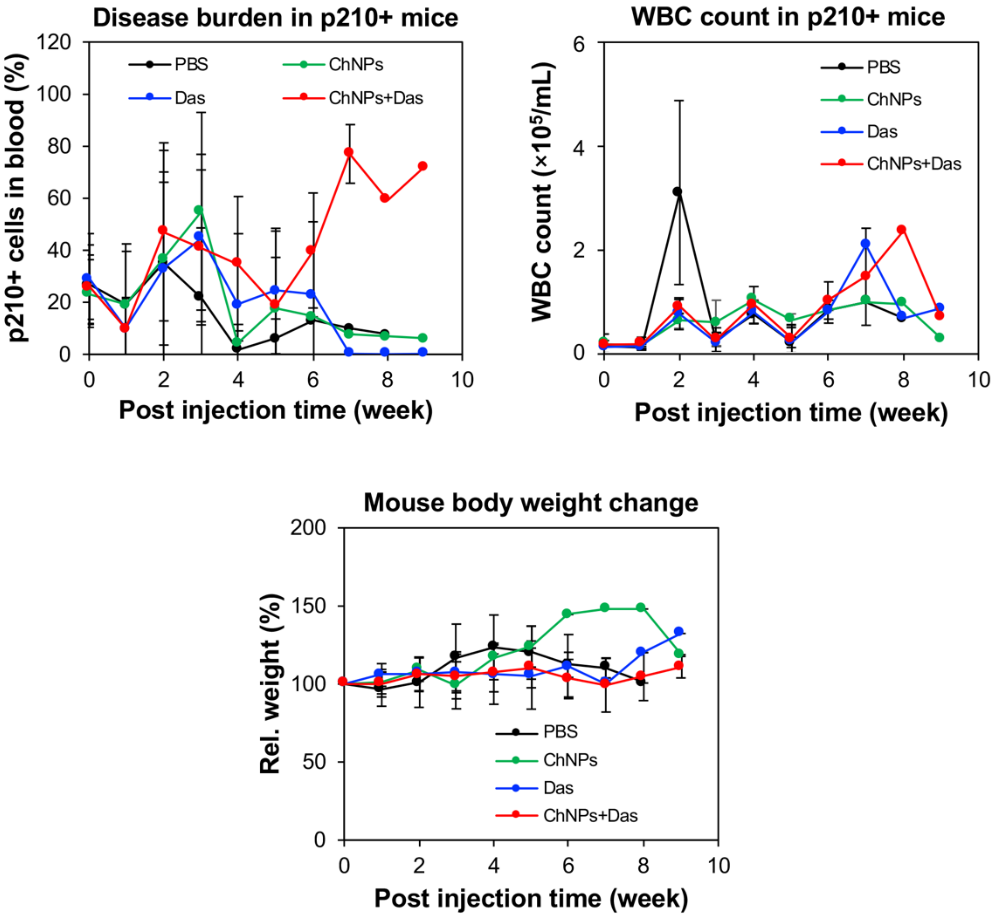

Figure S10. Disease burden, white blood cell (WBC) counts, and weights of diseased mice compared with weeks after treatment in mice with chronic phase BCR-ABL+ leukemia. Disease burden appeared higher over time in mice being treated with ChNPs + Das as mice with heavier disease burden were able to survive longer after treatment was removed, while other groups had reduced burden through losing mice in the group. All mice had similar WBC counts expect for PBS mice as they were going through blast crisis. Mice being treated with ChNPs were able to gain the most weight, but weight was largely steady for all groups.

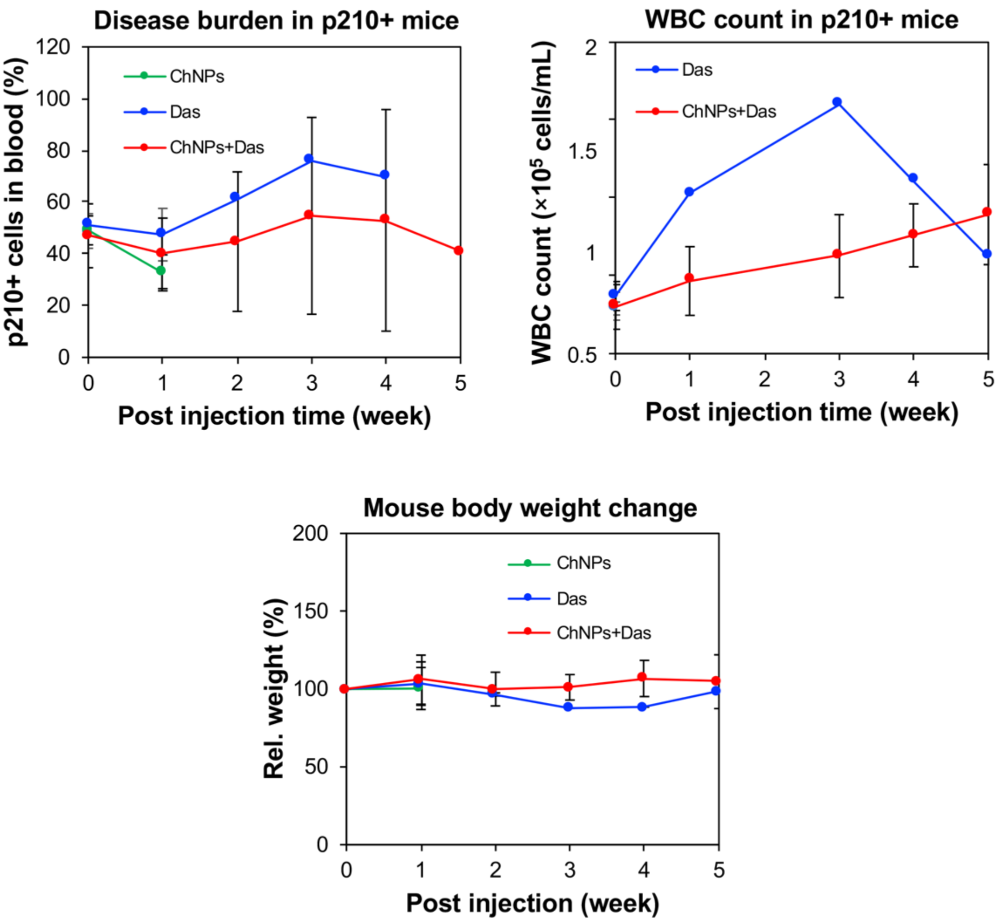

Figure S11. Disease burden, white blood cell (WBC) counts, and weights of diseased mice compared with weeks after treatment in mice with acute phase BCR-ABL+ leukemia. Disease burden was highest in the two dasatinib groups as mice with higher disease burden were able to live longer. WBC counts were higher in the dasatinib group compared to the ChNPs + Das group, indicating spreading disease. Body weight was consistent across treatment groups.

Table S1. Two-tailed student's *t*-test statistics for p210 (**Fig. 3A iii**)

	HEPES	Null AAV	Scr siRNA	Null AAV + Scr siRNA	BIM AAV	MCL-1 siRNA	BIM AAV + MCL-1 siRNA	Null/Scr ChNPs	Null/MCL-1 ChNPs	BIM/Scr ChNPs
Null AAV	0.15304									
Scr siRNA	0.26422	0.78157								
Null AAV + Scr siRNA	0.41491	0.12814	0.30509							
BIM AAV	0.47687	0.46278	0.54533	0.7827943						
MCL-1 siRNA	0.13521	0.59353	0.6375	0.6794987	0.83408					
BIM AAV + MCL-1 siRNA	0.18044	0.25179	0.38444	0.8339913	0.68772	0.34906				
Null/Scr ChNPs	0.08207	0.33305	0.53092	0.1649166	0.64367	0.78735	0.20803845			
Null/MCL-1 ChNPs	0.2428	0.85204	0.93093	0.4712769	0.61773	0.55285	0.41485507	0.61409		
BIM/Scr ChNPs	0.33513	0.61254	0.61513	0.7862966	0.91305	0.97045	0.65616349	0.81241	0.30605	
BIM/MCL-1 ChNPs	0.00017	0.03982	0.09016	0.0216902	0.0572	0.01738	0.01091418	0.01091	0.08295	0.01602

Table S2. Two-tailed student's *t*-test statistics for MiG (**Fig. 3A iv**)

	0 nM Das	5 nM Das	10 nM Das
0 nM Das + ChNPs	0.96237		
5 nM Das + ChNPs		0.48088	
10 nM Das + ChNPs			0.630422

Table S3. Two-tailed student's *t*-test statistics for p210 (**Fig. 3A iv**)

	0 nM Das	5 nM Das	10 nM Das
0 nM Das + ChNPs	0.00878		
5 nM Das + ChNPs		0.03002	
10 nM Das + ChNPs			0.019438

$p \leq 0.05$ $p \leq 0.05$ $p \leq 0.005$ $p \leq 0.001$

Table S4. Two-tailed student's *t*-test statistics for Ph+ CML (**Fig. 3B i**)

	HEPES	Null AAV	Scr siRNA	Null AAV + Scr siRNA	BIM AAV	MCL-1 siRNA	BIM AAV + MCL-1 siRNA	Null/Scr ChNPs	Null/MCL-1 ChNPs	BIM/Scr ChNPs	BIM/MCL-1 ChNPs	BIM/MCL-1 ChNPs + Das
Null AAV	0.37286											
Scr siRNA	0.15807	0.93588										
Null AAV + Scr siRNA	0.10385	0.15701	0.09587									
BIM AAV	0.27745	0.28633	0.12408	0.50568								
MCL-1 siRNA	0.08261	0.17016	0.04611	0.33219	0.84265							
BIM AAV + MCL-1 siRNA	0.5378	0.88647	0.91632	0.21665	0.36719	0.26035						
Null/Scr ChNPs	0.6151	0.19566	0.29605	0.43927	0.62491	0.53993	0.23458					
Null/MCL-1 ChNPs	0.01004	0.00315	0.00954	0.02373	0.02266	0.01555	0.00672	0.00084				
BIM/Scr ChNPs	0.00672	0.00645	0.00427	0.02202	0.01851	0.01145	0.02023	0.00438	0.05302			
BIM/MCL-1 ChNPs	0.00815	0.01112	0.0042	0.01882	0.00952	0.00874	0.02253	0.00384	0.76997	0.03731		
BIM/MCL-1 ChNPs + Das	0.00974	0.00405	0.00861	0.02184	0.01886	0.01406	0.0067	0.00016	0.01279	0.02405	0.23993	
Das	0.05595	0.06286	0.04788	0.06372	0.03656	0.04247	0.08497	0.01202	0.09677	0.31277	0.04303	0.04051

Table S5. Two-tailed student's *t*-test statistics for Ph+ CML BC (**Fig. 3B ii**)

	HEPES	Null AAV	Scr siRNA	Null AAV + Scr siRNA	BIM AAV	MCL-1 siRNA	BIM AAV + MCL-1 siRNA	Null/Scr ChNPs	Null/MCL-1 ChNPs	BIM/Scr ChNPs	BIM/MCL-1 ChNPs	BIM/MCL-1 ChNPs + Das
Null AAV	0.79527											
Scr siRNA	0.82388	0.7287										
Null AAV + Scr siRNA	0.65612	0.99925	0.4933									
BIM AAV	0.60398	0.41459	0.55168	0.3622267								
MCL-1 siRNA	0.47782	0.58427	0.62743	0.2472423	0.63761							
BIM AAV + MCL-1 siRNA	0.07064	0.87231	0.40723	0.8019707	0.4788	0.21193						
Null/Scr ChNPs	0.73931	0.89761	0.37586	0.68027	0.39347	0.16377	0.66896238					
Null/MCL-1 ChNPs	0.06489	0.45028	0.06111	0.1385819	0.25522	0.00194	0.28443014	0.05935				
BIM/Scr ChNPs	0.05142	0.48601	0.0279	0.2005163	0.25422	0.00355	0.26272418	0.07211	0.79819			
BIM/MCL-1 ChNPs	0.02169	0.06661	0.00585	0.0072109	0.05986	0.00295	0.04527416	0.000034	0.00928	0.01285		
BIM/MCL-1 ChNPs + Das	0.016	0.05482	0.00661	0.0049333	0.06007	0.00168	0.03348313	0.00056	0.00429	0.00891	0.10019	
Das	0.07694	0.43028	0.00389	0.162502	0.20744	0.01113	0.26912279	0.04159	0.45116	0.31066	0.01163	0.01096

Table S6. Two-tailed student's *t*-test statistics for Ph- AML (**Fig. 3B iii**)

	HEPES	Null AAV	Scr siRNA	Null AAV + Scr siRNA	BIM AAV	MCL-1 siRNA	BIM AAV + MCL-1 siRNA	Null/Scr ChNPs	Null/MCL-1 ChNPs	BIM/Scr ChNPs	BIM/MCL-1 ChNPs	BIM/MCL-1 ChNPs + Das
Null AAV	0.21671											
Scr siRNA	0.22706	0.23874										
Null AAV + Scr siRNA	0.52562	0.99164	0.05224									
BIM AAV	0.29412	0.97032	0.28838	0.9786762								
MCL-1 siRNA	0.20011	0.0057	0.0545	0.1559211	0.07009							
BIM AAV + MCL-1 siRNA	0.15434	0.30651	0.60145	0.4132965	0.22036	0.12634						
Null/Scr ChNPs	0.32521	0.40916	0.72848	0.1589389	0.39188	0.13706	0.71450082					
Null/MCL-1 ChNPs	0.0217	0.01028	0.00366	0.0056175	0.00615	0.00922	0.07455442	0.00681				
BIM/Scr ChNPs	0.22871	0.30808	0.98984	0.2223006	0.46945	0.05581	0.66451286	0.84273	0.0235			
BIM/MCL-1 ChNPs	0.00718	0.00198	0.00121	0.0028702	0.00113	0.00243	0.02961444	0.0068	0.01034	0.00804		
BIM/MCL-1 ChNPs + Das	0.0064	0.00152	0.00114	0.0027555	0.00103	0.00199	0.02902852	0.0072	0.01273	0.00714	0.11533	
Das	0.06115	0.03877	0.08243	0.0367278	0.0944	0.0145	0.71335674	0.26132	0.04554	0.00356	0.01139	0.00986

$p \leq 0.05$ $p \leq 0.05$ $p \leq 0.005$ $p \leq 0.001$

Table S7. Log-rank (Mantel-Cox) test statistics for chronic leukemia survival data (**Fig. 4B**)

	PBS	ChNPs	Das	ChNPs + Das
ChNPs	0.0938			
Das	0.0097	0.0798		
ChNPs + Das	0.0118	0.0162	NS	

Table S8. Log-rank (Mantel-Cox) test statistics for acute leukemia survival data (**Fig. 4B**)

	PBS	ChNPs	Das	ChNPs + Das
ChNPs	0.0091			
Das	0.0091	0.3893		
ChNPs + Das	0.0091	0.0825	0.4190	

Table S9. Two-tailed student's *t*-test statistics for pathology study (Liver, Day 11, **Fig. 4D**)

	Healthy	PBS	ChNPs	ChNPs + Das
PBS	* (0.0184)			
ChNPs	NS	NS		
ChNPs + Das	NS	* (0.0123)	NS	
Das	NS	* (0.0103)	NS	NS

Table S10. Two-tailed student's t-test statistics for pathology study (Liver, Day 47, **Fig. 4D**)

	Healthy	ChNPs + Das
ChNPs + Das	NS	
Das	** (0.0019)	** (0.0056)

Table S11. Two-tailed student's *t*-test statistics for pathology study (Spleen, Day 11, **Fig. 4D**)

	Healthy	PBS	ChNPs	ChNP + Das
PBS	*** (0.001)			
ChNPs	** (0.03)	NS		
ChNPs + Das	NS	*** (0.001)	*** (0.001)	
Das	NS	** (0.03)	** (0.012)	NS

Table S12. Two-tailed student's *t*-test statistics for Pathology study (Spleen, Day 47, **Fig. 4D**)

	Healthy	ChNPs + Das
ChNPs + Das	NS	
Das	* (0.0253)	* (0.047)

$p < 0.5$ $p < 0.05$ $p < 0.005$ $p < 0.001$
